# Analogy of transistor function with modulating photonic band gap in electromagnetically induced grating

**DOI:** 10.1038/srep13880

**Published:** 2015-09-09

**Authors:** Zhiguo Wang, Zakir Ullah, Mengqin Gao, Dan Zhang, Yiqi Zhang, Hong Gao, Yanpeng Zhang

**Affiliations:** 1 Key Laboratory for Physical Electronics and Devices of the Ministry of Education & Shaanxi Key Lab of Information Photonic Technique, Xi’an Jiaotong University, Xi’an 710049, China; 2 School of Science, Xi’an Jiaotong University, Xi’an 710049, China

## Abstract

Optical transistor is a device used to amplify and switch optical signals. Many researchers focus on replacing current computer components with optical equivalents, resulting in an optical digital computer system processing binary data. Electronic transistor is the fundamental building block of modern electronic devices. To replace electronic components with optical ones, an equivalent optical transistor is required. Here we compare the behavior of an optical transistor with the reflection from a photonic band gap structure in an electromagnetically induced transparency medium. A control signal is used to modulate the photonic band gap structure. Power variation of the control signal is used to provide an analogy between the reflection behavior caused by modulating the photonic band gap structure and the shifting of Q-point (Operation point) as well as amplification function of optical transistor. By means of the control signal, the switching function of optical transistor has also been realized. Such experimental schemes could have potential applications in making optical diode and optical transistor used in quantum information processing.

In analogy to their electronic counterparts, optical transistors and switches are required as fundamental building blocks for classical as well as quantum optical information processing^1^,^2^. In particular, an optical transistor and switch operated by a single photon stored in an atomic ensemble inside a cavity has recently been demonstrated^3^. In this article we will theoretically and experimentally demonstrate the analogy of an optical transistor function with the enhancement and suppression of multi-wave mixing process through the modulation of a photonic band gap structure. Four-wave mixing is a well-known nonlinear optical effect which can be enhanced in an electromagnetically-induced transparency (EIT) medium^4^,^5^,^6^. Four wave mixing^7^,^8^,^9^,^10^ and six wave mixing^11^ have been individually studied in multi-level atomic systems. By choosing appropriate atomic level schemes and driving fields, one can generate controllable nonlinearities with very interesting applications in designing novel nonlinear optical devices. This motivates the studies of enhanced higher-order nonlinear wave-mixing processes. Enhanced six-wave mixing via induced atomic coherence was experimentally observed in a four level inverted Y-type atomic system^12^. Such six-wave mixing signal can be made to even coexist, compete, and spatially interfere with the four-wave mixing signal in the same system^13^ by the assistance of EIT. The EIT-based nonlinear schemes can be driven both by traveling wave beams and standing wave beams. The large nonlinearity was obtained in an atomic system driven by two counter propagating coupling fields of the same frequency which form a standing wave^14^,^15^. Standing wave interacts with atomic coherent medium to result in an electromagnetically induced grating^16^,^17^. The electromagnetically induced grating possesses a photonic band gap structure as shown in Fig. 1(c). Such electromagnetically induced grating has potential applications in manipulation of light propagation to create tunable photonic band gap structures^18^,^19^,^20^. Moreover the relevant research can also be used to make optical diodes if the photonic crystal induced in the medium from periodic modulation of its optical properties is set into motion^21^.

In this letter, we report the optical response of rubidium (^85^*Rb*) atomic vapors driven by a standing wave coupling field and probe field, from which the optically controllable photonic band gap structure can be generated. We report an experimental and theoretical demonstration of the reflection from a photonic band gap structure along with probe transmission signal in EIT based inverted Y-type four level atomic system. We show four-wave mixing band gap signal (FWM BGS) can be suppressed and enhanced. The suppressed and enhanced FWM BGS can be used to provide an analogy with the switching and amplification functions of an optical transistor. The probe frequency detuning is used to find the optimal experimental conditions for the reflected band gap signal. The periodic energy levels generated by the standing wave under EIT condition are further modulated with the help of a control signal to exploit the photonic band gap structure and change the reflectivity. Manipulating the photonic band gap structure with the help of a control signal and changing power of the incident probe field are the two alternative ways used to change intensity of the reflection from a photonic band gap structure.

## Results

**Figure 1:**
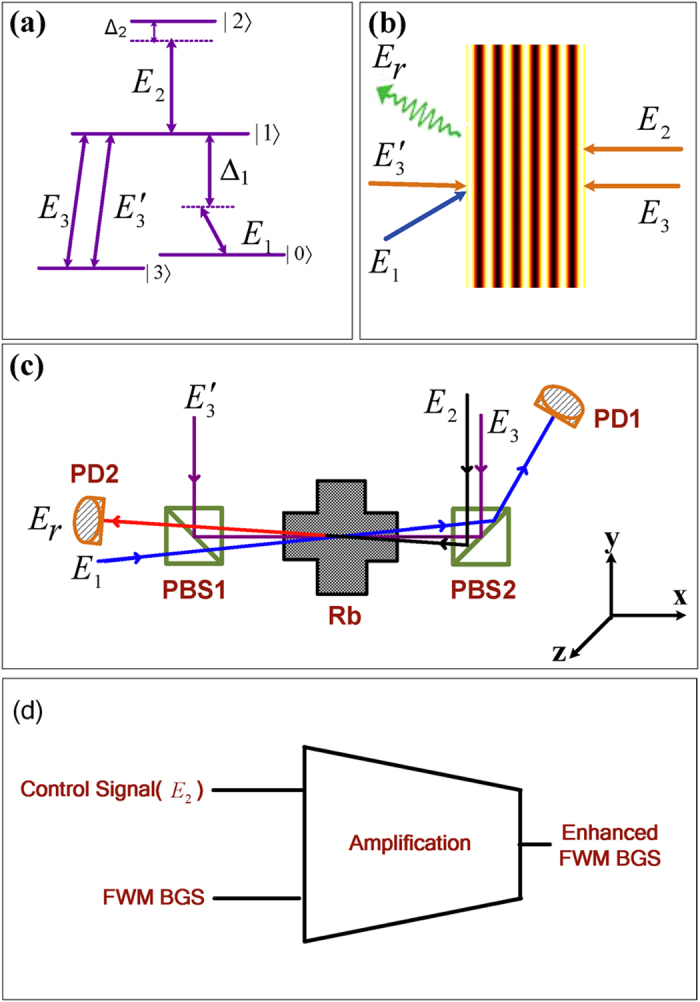
(**a**) Four-level energy system. (**b**) Schematic of an electromagnetically induced grating formed by two coupling beams *E*_3_ and 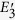. Together with the dressing field *E*_2_ and probe field *E*_1_, a dressed band gap signal *E*_*r*_ will be generated according to the phase-matching condition 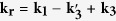. (**c**) The setup of our experiment. (**d**) Block diagram of the analogy of transistor amplification function.

The experiment was carried out in a cell contained rubidium (^*85*^*Rb*) atomic vapors for a simple inverted Y-type atomic system with four energy levels consisting of 5*S*_1/2_(*F* = 3)(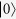), 5*P*_3/2_(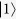), 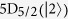 and 5*S*_1/2_(*F* = 2)(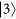) as shown in Fig. 1(a). The arrangement of the experimental setup and spatial alignment of laser beams *E*_*i*_(frequency*ω*_*i*_ and wave vector *k*_*i*_) is shown in Fig. 1(c). Incident probe field *E*_1_ with wavelength about 780.245 nm probes the transition 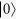 to 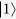. The counter-propagating fields *E*_3_ and 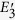 propagate through ^85^*Rb* vapors with wavelength about 780.238 nm, connecting the transition 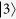 to 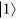. The dressing (or control) field *E*_*2*_ with wavelength of 775.978 nm drives an upper transition 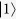 to 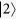. The coupling field ***E***_3s_ = *ŷ*[*E*_3_cos(*ω*_3_*t* − *k*_3_*x*) + *E′*_3_cos(*ω*_3_*t* + *k*_3_*x*)], composed of *E*_3_ and 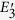, generates a standing wave. Rabi frequency of the coupling field is 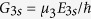. So we have 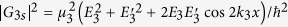. Interaction of standing wave with atomic coherent medium results into electromagnetically induced grating. Furthermore electromagnetically induced grating will lead to a photonic band gap structure as shown in Fig. 1(b). The probe field *E*_1_ propagates in the direction of 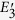 through the ^*85*^*Rb* vapors with approximately 0.3° angle between them. The control field *E*_2_ propagates in the opposite direction of 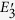 through ^*85*^*Rb* with approximately 0. 3° angle between them. *E*_*r*_ (The reflected band gap signal from the photonic band gap structure) and *E*_1_ is another pair of the counter-propagating beams, but with a small angle between them as shown in Fig. 1(c). Due to the small angle between *E*_1_ and 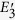, the geometry not only satisfies the phase-matching condition but also provides a convenient spatial separation of the applied laser and generated signal beams. Thus we can easily detect the generated beams which are highly directional^22^. The probe transmission signal and generated band gap signal are detected by photodiode detectors PD1 and PD2, respectively.

A block-diagram of the analogy of the behavior of modulating the photonic band gap structure with an optical transistor amplification function is shown in Fig. 1(d), where FWM BGS generated by *E*_1_, *E*_3_ and 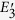 in the medium is analogous to the input signal *a*_*in*_ in the optical amplification experiment; enhanced FWM BGS is analogous to the output signal *a*_*out*_ and *G* is analogous to the gain factor of transistor. The mathematical model of this analogy is given as 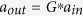. *E*_*2*_ is a control signal, power of which can be used to modulate the photonic band gap structure. This behavior is analogous to change the Q-point of electrical transistor.

Considering the time-dependent Schrödinger equation, using the perturbation chain 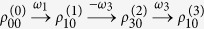 (i.e., Liouville pathways with perturbation theory^23^ and satisfying phase-matching condition) and rotating wave approximation, we can obtain a series of density matrix equations by using the way with combining the coupling method and the perturbation theory as follows


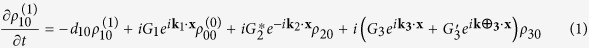



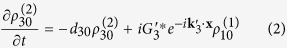



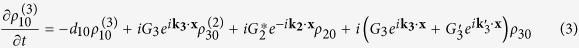



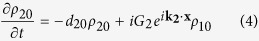



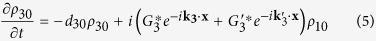


Where the superscript (0), (1), (2) or (3) express the perturbation order. 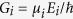 is the Rabi frequency with transition dipole moment 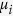. 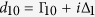, 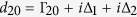, 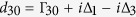, frequency detuning 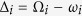 (Ω_*i*_ is the resonance frequency of the transition driven by *E*_*i*_). 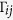 is transverse relaxation rate between 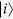 and 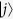. By solving Eqs. (1)–(5), 22, 23, 24, 25 with the steady state approximation and the condition 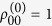 (which is reasonable since the probe field is always weak, compared with other fields), we finally obtain the first order and the third order density matrix elements 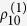 and 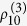. By similar method, with the perturbation chain 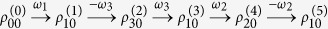, we can also obtain the fifth order density matrix element 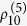 as follows


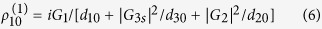



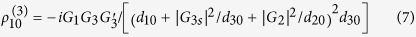



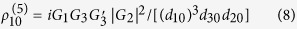


where 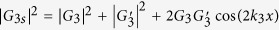. According to the relation 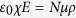, in which *N*, 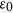 are the atoms density and dielectric constant respectively, corresponding susceptibilities can be obtained as follows:


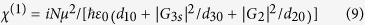



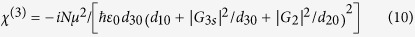



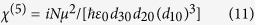


**Figure 2:**
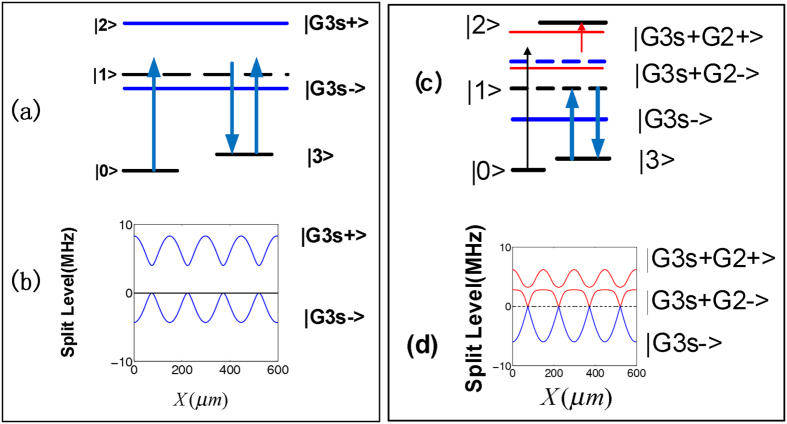
(**a**) The single dressed energy levels schematic diagrams and (**b**) the calculated single dressed period energy levels with changing Δ_3_. (**c**) The double dressed energy levels schematic diagrams and (**d**) the calculated double dressed periodic energy levels with changing Δ_2_.

The condition of generating the photonic band gap structure is that the medium is of periodic refractive index. In order to get the periodic refractive index, the susceptibility should be periodic according the relation of refractive index with susceptibility, i.e., 
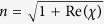
. To get periodic susceptibility we should generate the periodic energy levels structure. Hence, by introducing periodic standing wave field, we can obtain the periodic energy levels as shown in Fig. 2. In Fig. 2(a), level 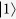 will be split into two dressed states 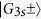 depending on Δ_3_ and 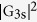. The two dressed states 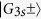 have the eigenvalues 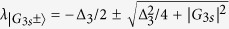. Since 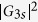 is periodic along x, so 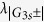 values are also periodic. Thus we can obtain the periodic energy levels as shown in Fig. 2(b). When the probe reaches two-photon resonance Δ_1_ − Δ_3_ = 0, the absorption will be suppressed, i.e. the probe transmission signal becomes strong. At the same time, the band gap signal will be suppressed correspondingly. Thus, we define Δ_1_ − Δ_3_ = 0 as the suppression condition. When *E*_2_ is turn on, 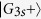 is further split into two dressed states 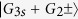 due to the second level dressing effect of *E*_2_. The two dressed states 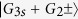 have the eigenvalues 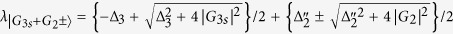 with 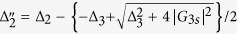. In our system, the normalized total susceptibility is 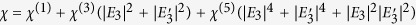, which determines the refractive index of the system according to 
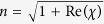
. For the system generating the band gap signal, the real part of susceptibility is periodic.

In order to estimate the reflection efficiency of band gap signal, we start with the wave equation in the following form^24^,


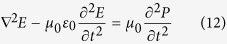


Where *P* is polarization of the medium given by 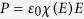 and 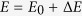 is the total field, where 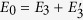 is the strong coupling fields and 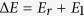 is the weak signal fields. Substituting *P* and *E* into Eq. (12) and using the slowly varying amplitude approximation 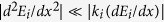, after equating the coefficients of the same exponential terms on both sides of the equation, we write the propagation equations for reflected band gap signal 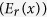 and probe transmission signal 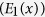 as follows^21^


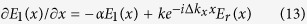



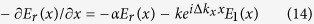



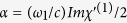
 is attenuation of the field due to the absorption of the medium and 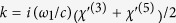 is the gain due to the nonlinear susceptibilities in which four-wave and six-wave mixing are considered. 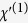, 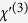 and 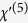 are the zero order coefficients from Fourier expansion of 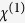, 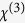 and 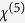, respectively. 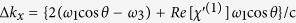 is the phase mismatch magnitude, in which *θ* is the angle between probe *E*_1_ and 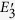. Equations (13) and (14) describe the mutual generation process of 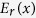 and 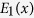 when they propagate inside the medium. For example, in Eq. (14), 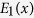 is generating field and 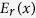 is the generated field, and vice versa for Eq. (13), while 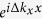 represents the generating efficiency. The generating efficiency will be high when the phase matching condition is satisfied (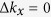). In order to estimate the efficiency of the reflected band gap signal and probe transmission signal, we solve Eqs. (13) and (14) as follows. We differentiate Eq. (13) with respect to *x* and simplify it by using Eq. (14) to get the following second order differential equation


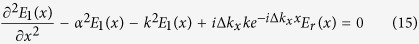


After eliminating 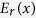 from Eqs. (13) and (15), we get the following equation


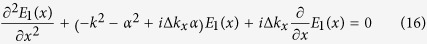


The general solution of Eq. (16) is 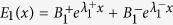. Next we substitute the value of 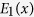 in Eq. (13) to get 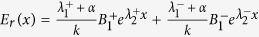. Where 
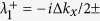
 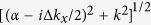 and 
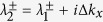
. We assume that length of the ^85^*Rb* sample is *d*_*x*_ and apply initial conditions 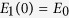 and 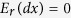 to get the values of 
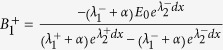
 and 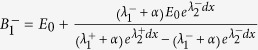. We define the reflection efficiency of band gap signal from the photonic band gap structure with respect to the incident probe field as


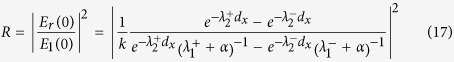


While the probe transmission efficiency across the medium with respect to the incident probe field is defined as


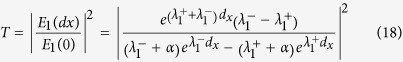


**Figure 3:**
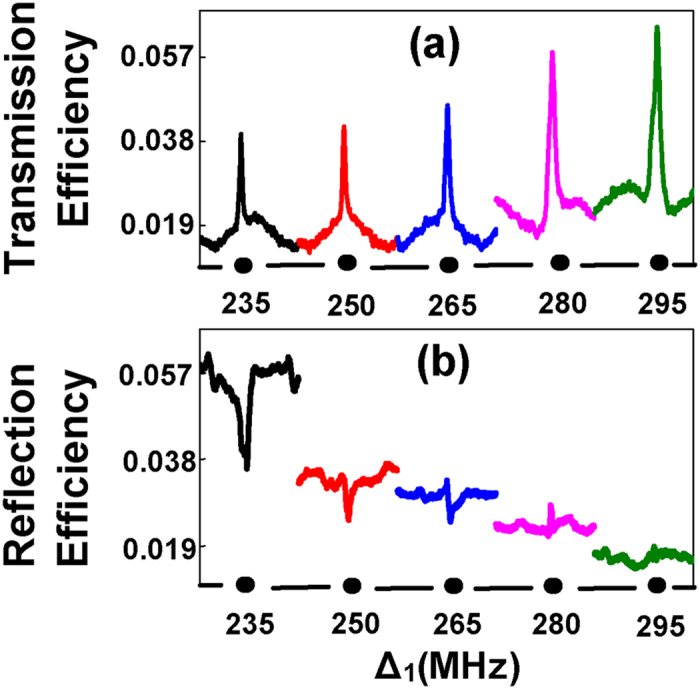
Measured (a) efficiency of probe transmission signal and (b) efficiency of reflected band gap signal (*E*_*r*_) versus Δ_2_, when we select five different discrete values of Δ_1_ as black (235 MHz), red (250 MHz), blue (265 MHz), pink (280 MHz) and green (295 MHz) and Δ_3_ = 230 MHz. The discrete X-axis consist of solid black circles shows the different discrete designated Δ_1_ for each sub-curve. Total scanning frequency range of Δ_2_ in each sub-curve is about 240 MHz.

First, we observe the variations in the intensities of band gap signal and probe transmission signal by scanning Δ_2_ at different discrete values of Δ_1_ as shown in Fig. 3. Note that variations in the intensities discussed here are displayed by the efficiencies of probe transmission signal and reflected band gap signal in the following figures according to the above theory. In Fig. 3(a), each sub curve’s peak is the dressed probe transmission signal induced by the second-level dressing effect of *E*_2_, which meets the condition 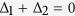 according to the dressing term 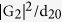 of Eq. (6). The smallest peak appears at Δ_1_ = Δ_3_ because of the strongest cascaded interaction between *E*_3_ and *E*_2_ as depicted by the doubly dressed term 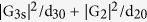 in Eq. (6). In Fig. 3(b) the baselines show the intensity of FWM BGS, which is the reflection from photonic band gap structure. The dip in each sub curve shows that FWM BGS is suppressed while the peak within each sub curve represents that FWM BGS is enhanced. It is worth mentioning that, in the case of scanning the dressing frequency detuning Δ_2_, the suppression and enhancement of FWM BGS are caused by the same dressing term 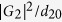 of 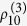 in Eq. (7), but at different positions. The suppression of FWM BGS occurs at the dark state position 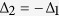 while the enhancement occurs at the bright state position 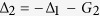. The six-wave mixing band gap signal whose efficiency is given by *R* in Eq. (17) with 
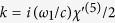
 locates at 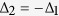 according to 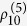 in Eq. (8), which is so weak that it is submerged in the suppression dip of FWM BGS. The expansion of 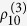 gives us very interesting information about band gap signal, which is 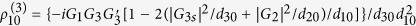. The first term is related to the intensity of baseline of FWM BGS without the dressing effect while the last term results in the suppression dip of FWM BGS at the dark state position and the enhancement peak of FWM BGS at the bright state position with the dressing effect. Enhancement of FWM BGS is used to demonstrate the analogy of an optical transistor function with the behavior of modulating the reflected band gap signal. With Δ_1_ changing from small to large values, enhancement peak of FWM BGS first becomes obviously large and then at very larger detuning it becomes small again. Figure 3(b) shows that the probe detuning can be used to find the optimal conditions for the reflected band gap signal. This information can be used to give the analogy of the enhancement and suppression of the reflected band gap signal with optical amplification and switching. Maximum enhancement of band gap signal occurs at particular detuning (Δ_1_ = 280 MHz), while the maximum suppression occurs at Δ_1_ = 235 MHz, as shown in Fig. 3(b). 235 MHz and 280 MHz are the optimal values of Δ_1_ which are used to demonstrate the optical switch and amplification functions.

**Figure 4:**
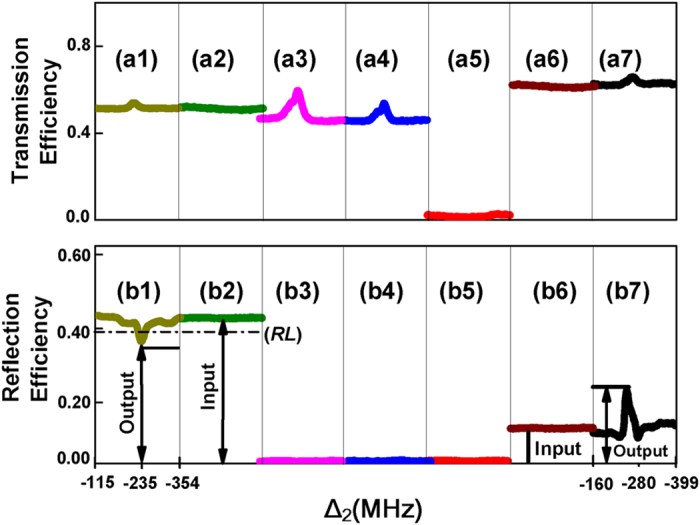
Measured (a) efficiency of probe transmission signal and (b) efficiency of reflected band gap signal (*E*_*r*_) versus Δ_2_, with Δ_3_ = 230 MHz when different beams are blocked. First, when Δ_1_ = 235 MHz, (a1) (b1) no beam blocked, (a2) (b2) *E*_2_ blocked, RL represents reference level; Next, when Δ_1_ = 280 MHz, (a3) (b3) *E*_3_ blocked, (a4) (b4) 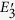 blocked, (a5) (b5) *E*_1_ blocked, (a6) (b6) *E*_2_ blocked and (a7) (b7) no beam blocked. Total scanning frequency range of Δ_2_ in each sub-curve is about 240 MHz.

Next, we observe the variations in the intensities of probe transmission signal and band gap signal versus dressing frequency detuning Δ_2_ by blocking different beams at two optimal values of Δ_1_ as shown in Fig. 4. Based on the values of Δ_1_, we classify the signals in two different groups. First, Fig. 4(a1–b1) shows probe transmission signal and band gap signal with no blocking and blocking *E*_2_ when Δ_1_ is set as 235 MHz. In Fig. 4(a1), when all the beams are turned on, there is a peak in the probe transmission signal because of the dressing term 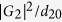 of 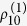 in Eq. (6) which is suppressed by 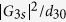. The efficiency of the probe transmission signal is measured by *T* in Eq. (18). In Fig. 4(a2), the peak in the probe transmission signal disappears by blocking *E*_2_ because of the absence of dressing term 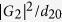 of 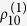 in Eq. (6). At the moment, the height of the straight line represents the intensity of probe transmission signal caused by *E*_3*s*_ according to 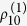, which is of the same intensity with the baseline of sub curve in Fig. 4(a1). To demonstrate the switching function of a transistor, the value of Δ_1_ should be set as about 235 MHz. At the same time, we define the intensity of the band gap signal lower than reference level (RL) as OFF-State while the intensity higher than reference level as ON-State of the switch. Two extreme values 0 mW and 21 mW of the power of control signal (*P*_2_) are used to turn on or off the switch. The two extreme values of *P*_2_ are analogous to the digital values of gate voltage or base current in the case of MOSFET and BJT respectively. In Fig. 4(b2), when *P*_2_ is set to 0 mW, the band gap signal is analogous to the input signal of a transistor. This band gap signal is FWM BGS generated by *E*_1_, *E*_3_ and 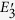 without *E*_2_ according to 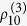 of Eq. (7) in the medium. When *P*_2_ is set to 0 mW, the input signal directly passes to the output (the detector PD2 (Fig. 1(c))). Since the output signal intensity is higher than reference level, the switch is ON-State. When *P*_2_ is set as 21 mW, the baseline in Fig. 4(b1) has the same intensity with the band gap signal in Fig. 4(b2). The dip shows that FWM BGS is suppressed because of the dressing term 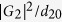 of 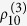 in Eq. (7). Physically, due to the modulation effect of *E*_2_ on the photonic band gap structure at Δ_2_ = −Δ_1_, the reflected FWM BGS will get weak. At the moment, the output signal intensity (which is given by the lowest point of curve in Fig. 4(b1)) at the detector PD2 (Fig. 1(c)) is lower than the reference level and the switch is OFF-State. Next, from Fig. 4(a3–b3) to Fig. 4(a7–b7), we observe the variations of probe transmission signal and reflected band gap signal by blocking *E*_3_, blocking 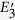, blocking *E*_1_, blocking *E*_2_ and no blocking with Δ_1_ = 280 Mhz from left to right, respectively. The results in Fig. 4(a6 are similar with the ones in Fig. 4(a1. In Fig. 4(a3, intensities of the peaks in the probe transmission signal increases by blocking *E*_3_ and 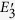, respectively, compared with Fig. 4(a7) (no blocking). This is because of the decreasing suppression effect of 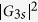 in the cascaded dressing term 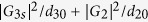 of 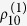 in Eq. (6). Intensity of the probe transmission signal will become zero by blocking the incident probe beam (*E*_1_) because of *G*_1_ is zero in Eq. (6) as shown in Fig. 4(a5). The reflected band gap signal can be enhanced with increasing *P*_2_ when Δ_1_ is set as about 280 MHz as shown Fig. 4(b7), whose efficiency is measured by *R* in Eq. (17). This behavior is analogous to the amplification function of transistor. To demonstrate the analogy of the enhancement of the band gap signal with amplification function of a transistor, we need set Δ_1_ as about 280 MHz. Compared with Fig. 4(b7) (no blocking), the enhancement peaks in the band gap signal disappear by blocking 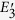, *E*_3_, *E*_1_ or *E*_2_, respectively, as shown in Fig. 4(b3–b6). As shown in Fig. 4(b3, when any one of 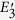 or *E*_3_ is blocked, the photonic band gap structure is deformed and therefore the reflected band gap signal disappears. In Fig. 4(b5), when the incident probe beam is blocked, there is still no reflection because of the absence of incident signal source *E*_1_ according to 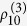 in Eq. (7) although the photonic band gap structure is there. When *E*_2_ is turned off, Fig. 4(b6) shows the FWM BGS generated by *E*_1_, *E*_3_ and *E*_3_ according to 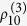 of Eq. (7). The FWM BGS is analogous to the input signal in the optical amplification experiment. When *E*_2_ is turned on, the FWM BGS can be obviously enhanced as shown in Fig. 4(b7). Physically, at the large detuning Δ_1_ = 280 Mhz, due to the modulation effect of *E*_2_ on the photonic band gap structure at 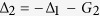, the reflected FWM BGS will become strong. The highest point of peak in Fig. 4(b7) gives the amplified intensity of the band gap signal, which is the output in the optical amplification experiment. Furthermore, the baseline in Fig. 4(b7) has the same intensity with the band gap signal in Fig. 4(b6), which can also be viewed as the input signal in the optical amplification experiment. It is clear from the above discussion, to operate the system as a switch, we need set Δ_1_ as about 235 MHz; while to operate it as an amplifier, we need set Δ_1_ as about 280 MHz and make the power of incident probe smaller at the same time.

**Figure 5:**
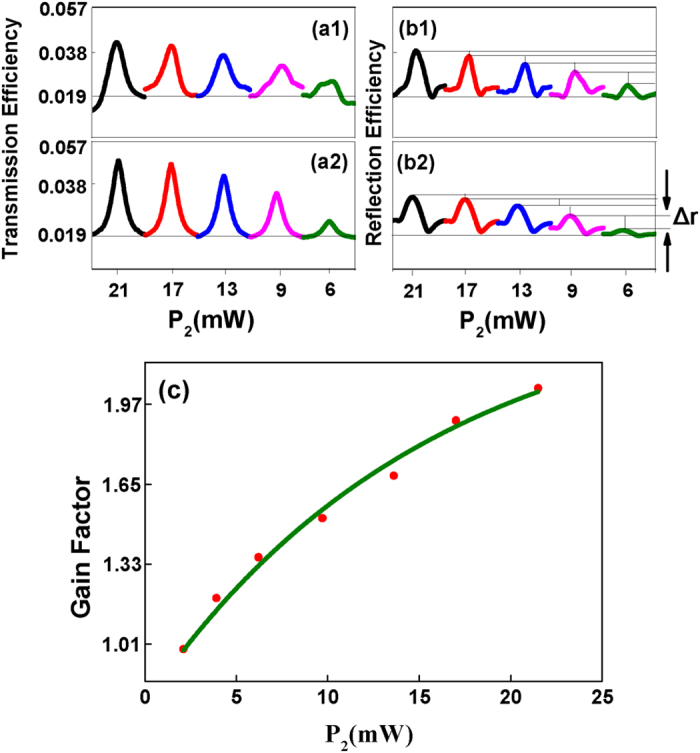
Measured (a1) efficiency of probe transmission signal and (b1) efficiency of enhanced four wave mixing band gap signal (*E*_*r*_) versus Δ_2_ from 160 MHz to 400 MHz with Δ_3_ = 230 MHz and Δ_1_ = 280 MHz, when we set the power of *E*_*2*_ *(P*_*2*_) as (Black) 21.5 mW, (Red) 17.0 mW, (Blue) 13.6 mW, (Pink) 9.7 mW, (Green) and 6.2 mW, respectively. (a2) and (b2) are the theoretical calculations of (a1) and (b1), respectively. (**c**) Gain factor of the amplifier versus *P*_*2*_. (Solid circles are the experimental data points, while the solid line is theoretical fitting result).

Next, we further demonstrate the analogy of modulating the band gap signal with the optical transistor amplification function with the power dependences of probe transmission signal and band gap signal versus Δ_2_. Variations in the two types of signals are shown from right to left with increasing power of *E*_2_(*P*_2_) as shown in Fig. 5(a1–b1). In Fig. 5(a1), the peak in each baseline shows the enhancement of the probe transmission signal induced by the dressing effect of *E*_2_. The efficiency of the probe transmission signal is given by *T* in Eq. (18). The dressing term 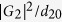 of 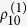 has an enhancement effect on the probe transmission signal. Changing *P*_*2*_ from small to large values, the peak becomes higher with the increasing dressing effect of 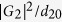 in 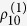 of Eq. (6). In Fig. 5(b1), the baseline of each sub curve shows intensity of FWM BGS generated by *E*_1_, *E*_3_ and 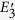 which is analogous to the input signal in the optical amplification experiment according to the discussion about Fig. 4(b6. Dips at Δ_2_ = −Δ_1_ show the suppression of reflected FWM BGS because of the dressing effect of *E*_2_ according to the dressing term 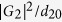 in 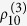 of Eq. (7). The efficiency of FWM BGS is measured by *R* in Eq.(17) with 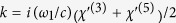. The dip is shallow at small value of power and becomes deeper with increasing *P*_2_ due to the enhanced dressing effect of *E*_2_ from right to left in Fig. 5(b1). Peaks show the enhancement of FWM BGS, the efficiency of which is also given by *R* in Eq. (17) with 
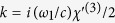
. The highest point on each sub curve’s peak in Fig. 5(b1) is the output in the optical amplification experiment. The intensity of band gap signal increases with increasing *P*_2_. It is important to mention here that 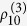 in Eq. (7) shows that enhancement of FWM BGS at 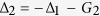 is because of the dressing term 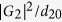. Interestingly, the dressing tem 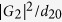 is common to both 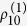 and 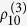. Therefore, the band gap signal with the higher intensity corresponds to the stronger probe transmission signal as shown in Fig. 5. Thus it is important to measure the probe transmission signal because of its strong relation with the band gap signal. Here we give an analogy of the amplification function of the transistor with modulating the ban gap signal. This analogy of the amplification function is demonstrated by two alternative ways which are modulating photonic band gap structure and changing the power of the incident probe field (*E*_*1*_). Here we modulate the photonic band gap structure by changing the power of control signal (*P*_2_) to get the amplified output signal, while powers of the incident probe and coupling fields are held constant, i.e. the FWM BGS generated by the probe and coupling fields is constant, which is viewed as the input here. As mentioned earlier band gap signal is the reflection of a controllable photonic band gap structure. Therefore the intensity of the reflection can be changed by controlling the photonic band gap structure or by changing power of the incident probe field (*E*_1_). The photonic band gap structure can be controlled by changing *P*_2_. As shown in Fig. 2(d), *P*_2_ further modulates the periodic energy levels to change the generated photonic band gap structure. Like the electrical transistors, changing *P*_2_ is analogous to changing biasing of electrical transistor. When the biasing of a transistor is changed, its operation point (Q-point) is shifted. As shown in Fig. 5(c), the gradient of gain factor (the ratio of the amplified band gap signal intensity (the highest point in the peak) to the original FWM BGS intensity (the baseline)) versus *P*_2_ curve is gradually decreasing as the amplification of band gap signal tends toward saturation. Similarly the relative amount of amplification (Δ*r*) also decreases with *P*_2_ increasing for a fixed input signal as the bang gap signal intensity reaches the saturation region as shown in Fig. 5(b1). This behavior is similar to the variation of amplifier output by changing Q-point or biasing of a transistor. Figure 5(a2–b2) shows the theoretical calculations of Fig. 5(a1–b1), which are in well agreement with the experimental results.

**Figure 6:**
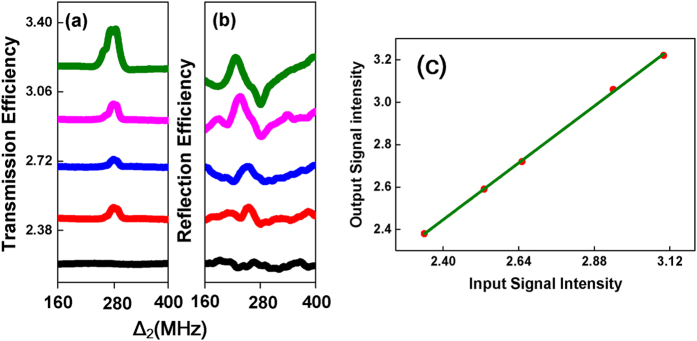
Measured (a) efficiency of probe transmission signal, (b) efficiency of enhanced four wave mixing band gap signal (*E*_*r*_) versus Δ_2_ with Δ_3_ = 230 MHz, Δ_1_ = 280 MHz and *P*_2_ = 3 mW, when we set the power of *E*_1_(*P*_1_) from top to bottom as (1) 2.68 mW, (2) 2.01 mW, (3) 1.10 mW, (4) 0.62 mW, and (5) 0.43 mW, respectively. (**c**) Output signal intensity (enhanced FWM BGS whose intensity is the one of the highest point on the peak of each sub curve in (**b**)) versus input signal intensity(FWM BGS whose intensity is the one of the baseline in each sub curve in (**b**)).

Finally, we change the power of *E*_1_(*P*_1_) and observe the variations in probe transmission signal and band gap signal versus Δ_2_. Power dependences of the two types of signals are shown from top to bottom with decreasing power of *E*_1_(*P*_1_) as shown in Fig. 6(a,b). The intensity of the probe transmission signal decreases with decreasing power of *E*_1_ according to 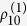 in Eq. (6). Peaks in Fig. 6(a) become smaller from top to bottom with the decreasing power of *E*_1_ because of *G*_1_ in Eq. (6). In Fig. 6(b), the baseline of the signal represents the intensity of the FWM BGS generated by *E*_1_, *E*_3_ and 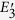 which is analogous to the input here according to the discussion about Fig. 4(b6. Dips show the suppression of the FWM BGS because of the dressing term 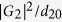 of 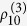 in Eq. (7). The suppression is further modulated by changing *P*_1_. The dip becomes shallow from top to bottom by changing *P*_1_ from large to small values. Peaks in the baseline show the enhancement of the FWM BGS. The highest point on the peak gives the intensity of the amplified band gap signal, which is the output of the amplifier. Compared to the previous case where we changed the power of *E*_2_, here the band gap signal intensity is changed by changing *P*_1_. Since *E*_1_ is the generating field for FWM BGS which is viewed as the input in the optical amplification experiment, the intensities of input signals increase as increasing *P*_1_, as shown by x coordinate values of solid circles in Fig. 6(c) which are obtained by measuring the intensities of baselines of Fig. 6(b). As a result, the output intensities (the highest points on the peaks) change in proportion to the input intensities when the power of the control signal (*E*_2_) is fixed as shown by y coordinate values of solid circles in Fig. 6(c) which are obtained by measuring the intensities of highest points of peaks in Fig. 6(b). The gradient of curve in Fig. 6(c) is constant, which is analogous to the behavior of electrical transistor operated at a fixed Q-point with a constant gain (*G*) due to the fixed power of the control signal (*E*_2_) which decides the Q-point.

## Discussion

In summary, the double-dressed probe transmission signal and band gap signal are compared for the first time to deeply comprehend the double-dressing effect on the photonic band gap structure. We experimentally and theoretically demonstrated that, probe transmission signal and band gap signal can be manipulated by multiple parameters like changing power and frequency detuning. We demonstrate the analogy between switching and amplification function of the transistor with modulating the reflected band gap signal. Such research could find its applications in optical diodes and transistors which are used in quantum information processing.

## Methods

In our experiment, there are four laser beams generated by three external cavity diode lasers (ECDL) with line width of less than or equal to 1 MHz. The probe laser beam ***E***_1_ is from an ECDL with a horizontal polarization. The two coupling laser beams ***E***_3_ and 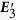 with a vertical polarization are split from another ECDL. The dressing laser beam ***E***_2_ with a vertical polarization is from the third ECDL. The intensity of probe beam *E*_*1*_ is the only weak laser beam while other laser beams are strong. The powers of ***E***_1_, ***E***_3_ and 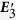 are 2.1 mW, 13.2 mW and 8.4 mW, respectively. And *P*_2_ are set as 21 mW in the experiment of changing probe frequency detuning. The atomic vapor cell has the typical density of 2 × 10^11^ cm^−3^. We measure the probe transmission signal and band gap signal in the inverted Y-type four level atomic system which can be dressed by fields ***E***_3_(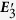), ***E***_2_. The four and six wave mixing band gap signals satisfy the phase-matching conditions 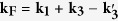 and 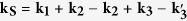, respectively.

## Additional Information

**How to cite this article**: Wang, Z. *et al.* Analogy of transistor function with modulating photonic band gap in electromagnetically induced grating. *Sci. Rep.* **5**, 13880; doi: 10.1038/srep13880 (2015).
